# Hepatitis B spliced protein (HBSP) promotes the carcinogenic effects of benzo [alpha] pyrene by interacting with microsomal epoxide hydrolase and enhancing its hydrolysis activity

**DOI:** 10.1186/1471-2407-14-282

**Published:** 2014-04-23

**Authors:** Jin-Yan Chen, Wan-Nan Chen, Bo-Yan Jiao, Wan-Song Lin, Yun-Li Wu, Ling-Ling Liu, Xu Lin

**Affiliations:** 1 Key Laboratory of Ministry of Education for Gastrointestinal Cancer, Research Center of Molecular Medicine, Fujian Medical University, Fuzhou, Fujian 350004, P.R. China; 2 Fujian Academy of Medical Sciences, Fuzhou, Fujian 350001, P.R. China; 3 Key Laboratory of Tumor Microbiology, Department of Medical Microbiology, Fujian Medical University, Fuzhou, Fujian 350004, P.R. China

**Keywords:** Hepatitis B virus, RNA splicing, Benzo[alpha]pyrene, Hepatocellular carcinoma

## Abstract

**Background:**

The risk of hepatocellular carcinoma (HCC) increases in chronic hepatitis B surface antigen (HBsAg) carriers who often have concomitant increase in the levels of benzo[alpha]pyrene-7,8-diol-9,10-epoxide(±) (BPDE)-DNA adduct in liver tissues, suggesting a possible co-carcinogenesis of Hepatitis B virus (HBV) and benzo[alpha]pyrene in HCC; however the exact mechanisms involved are unclear.

**Methods:**

The interaction between hepatitis B spliced protein (HBSP) and microsomal epoxide hydrolase (mEH) was confirmed using GST pull-down, co-immunoprecipitation and mammalian two-hybrid assay; the effects of HBSP on mEH-mediated B[alpha]P metabolism was examined by high performance liquid chromatography (HPLC); and the influences of HBSP on B[alpha]P carcinogenicity were evaluated by bromodeoxyuridine cell proliferation, anchorage-independent growth and tumor xenograft.

**Results:**

HBSP could interact with mEH *in vitro* and *in vivo*, and this interaction was mediated by the N terminal 47 amino acid residues of HBSP. HBSP could greatly enhance the hydrolysis activity of mEH in cell-free mouse liver microsomes, thus accelerating the metabolism of benzo[alpha]pyrene to produce more ultimate carcinnogen, BPDE, and this effect of HBSP requires the intact HBSP molecule. Expression of HBSP significantly increased the formation of BPDE-DNA adduct in benzo[alpha]pyrene-treated Huh-7 hepatoma cells, and this enhancement was blocked by knockdown of mEH. HBSP could enhance the cell proliferation, accelerate the G1/S transition, and promote cell transformation and tumorigenesis of B[alpha]P-treated Huh-7 hepatoma cells.

**Conclusions:**

Our results demonstrated that HBSP could promote carcinogenic effects of B[alpha]P by interacting with mEH and enhancing its hydrolysis activity.

## Background

Hepatocellular carcinoma (HCC) is the fifth most common cancer and the third leading cause of cancer death worldwide [1]. Hepatocarcinogenesis is a slow multistep and multifactorial process involving a progressive accumulation of changes at gene and protein level [2]. Chronic hepatitis B virus (HBV) infection is a major risk factor for HCC in the endemic areas. Several lines of evidences suggested that a synergistic interaction between environmental carcinogens and HBV-carcinogens may play a critical role in the carcinogenesis of HCC [3,4].

HBV is a small enveloped hepatotropic virus with a partially double-stranded DNA genome of approximately 3.2 kb in length [5]. In addition to the immune response to the viral proteins, which is considered to play a major role in the liver disease outcome, some HBV proteins also directly participate in chronic hepatitis and HCC, among which transcription activators of X protein (HBx) [6,7] and truncated middle surface protein (MHBst) have been extensively studied [8]. In the past decade, a novel hepatitis B spliced protein (HBSP) encoded by a 2.2 kb singly spliced defective HBV genome (spliced between positions 2447 nt and 489 nt) has been detected in the liver tissues and the serum from patients with hepatitis B [9,10]. HBSP has been shown to play an important role in hepatopathogenesis [11-13], yet the exact mechanisms remain to be fully elucidated.

Polycyclic aromatic hydrocarbons (PAHs) are ubiquitous environmental pollutants [3], exposure to which causes many cancers mostly mediating through the PAHs’ reactive metabolites, dihydrodiol epoxides [14]. Benzo[α]pyrene (B[alpha]P) is a best characterized PAH compound and is considered to be an indirect genotoxin. Its carcinogenic and mutagenic effects are manifested after being converted in vivo into a vicinal B[alpha]P-7,8-diol-9,10-epoxide (BPDE) [15,16]. In male infant mice, exposure to B[alpha]P induces liver tumors [17]. In addition, epidemicological studies have shown that the risk for developing HCC increased dramatically in those with the combination of higher BPDE-DNA adducts and HBV infection [3,4], suggesting the possible role of HBV-B[alpha]P interaction in hepatocarcinogenesis.

Microsomal epoxide hydrolase (mEH) plays a pivotal role in B[alpha]P conversion by hydration of B[alpha]P-7,8-oxide to B[alpha]P-7,8-diol, an important intermediate molecule of B[alpha]P metabolism [18,19]. The critical role of mEH bioactivation in PAH-induced carcinogenesis was demonstrated in EPHX1 (coding for mEH) null mice which were completely resistant to the tumorigenic effects of dimethylbenz[alpha]anthracene in a complete carcinogenesis assay [20]. In our previous study by a yeast two-hybrid screening [21], mEH was identified as a specific binding partner for HBSP from a human liver cDNA library. In this study, complex formation between HBSP and mEH under both cell-free and intracellular conditions was further confirmed, and the effects of HBSP on mEH-mediated B[alpha]P metabolism and the carcinogenic effects of B[alpha]P were evaluated. The results demonstrated that HBSP could promote carcinogenic effects of B[alpha]P by interacting with mEH and enhancing its hydrolysis activity.

## Methods

### Plasmid constructs

Vector pCMVTNT-EPHX1 used for in vitro translation of microsomal epoxide hydrolase (mEH) was constructed by inserting of EPHX1 gene (GeneBank Accession No. NM_000120) cDNA into pCMVTNT (Promega, Madison, WI, USA) between the Xho I and Kpn I sites, EPHX1 gene cDNA was amplified by reverse transcription polymerase chain reaction (RT-PCR) from the total RNA isolated from Huh-7 hepatoma cells. The primers used were: forward primer, 5′- CCGCTCGAGGCCACCATGTGGCTAGAAATCCTCCTCACT-3′; reverse primer, 5′-CGGGG TACCTCATTGCCGCTCCAGCAC-3′. pACT-EPHX1_353–455_ coding for 353–455 amino acids of mEH was generated by an in-frame insertion of PCR amplified fragment using screened cDNA library prey plasmid as a template into pACT between the Sal I and Not I sites (encodes a herpes simplex virus type 1 VP16 protein, Promega). The primers used were: forward primer: 5′-ACGCGTCGACTTGACCTGCTGACCAAC-3′; reverse primer: 5′-ATAAGAATGCGGCCGC TCATTGCCGCTCCAGCAC-3′. pGEX-HBSP coding for GST-HBSP protein, pBIND-HBSP, pBIND-HBSP1-47 and pBIND-HBSP48-111, which respectively codes for GAL4 DNA-binding domain fused full length, N terminal 47 amino acids and C terminal 64 amino acids of HBSP, were described previously [21].

### GST pull-down assay

E. coli Rosetta (DE3) (Novagen, Madison, Wisconsin, USA) was transformed with pGEX-HBSP, and the expression of GST-fused HBSP was induced with 0.5 mM isopropy l-β-D-thiogalactopyranoside (IPTG, Merck, Darmstadt, Germany) for 3 h at 28°C. The cells were harvested and suspended in phosphatate-buffered saline (PBS) containing 5 mM DTT and protease inhibitor cocktail (Roche Diagnostics, Mannheim, Germany). The cells were then disrupted by sonication. After centrifugation, the glutathione-Sepharose 4B beads (GE Healthcare, Uppsala, Sweden) were added to the supernatants and incubated overnight at 4°C. Then the glutathione-Sepharose 4B beads were washed three times with PBS containing 5 mM DTT and protease inhibitor cocktail (Calbiochem, La Jolla, CA). ^35^S-Labeled mEH protein was prepared using the TNT T7 Coupled Reticulocyte Lysate System (Promega) by adding 2 μg of pCMVTNT-EPHX1 and 50 μCi of ^35^S-methionine (Amersham Pharmacia Biotech, Piscataway, NJ, USA). For GST pull-down assay, ^35^S-labeled mEH was added to the GST recombinant proteins and glutathione-Sepharose 4B beads, and incubated overnight at 4°C. Beads were washed three times with 1% Triton X-100 in PBS, re-suspended and subjected to 12% sodium dodecyl sulphate-polyacrylamide gel electrophoresis (SDS-PAGE). The presence of ^35^S-mEH was detected by autoradiography.

### Co-immunoprecipitation (Co-IP) assay

A total of 4 × 10^6^ Huh-7 hepatoma cells in a 10 cm dish were transfected with 12 μg each of the constructs pBIND-HBSP, pBIND-HBSP_1–47_, pBIND-HBSP_48–111_ or pBIND. Forty-eight hours after transfection, the cells were washed three times with PBS and lysed using RIPA lysis buffer (Pierce, Rockford, IL, USA) containing a proteinase inhibitor cocktail (Roche Diagnsotics). The soluble proteins were pre-cleared with 100 μL of a 50% slurry of protein A agarose (Invitrogen, Carlsbad, CA, USA), and then the clear lysates were mixed with 2 μg of goat polyclonal anti-mEH IgG (Santa Cruz Biotechnology, Santa Cruz, CA, USA) and 100 μL of a 50% slurry of protein A agarose. The immunoprecipitated complexes were washed with lysis buffer and then analyzed by 12% SDS-PAGE and western blot, using specific antibodies including anti-GAL4BD monoclonal antibody (1:4000 dilution, Clontech, Palo Alto, CA, USA) and anti-mEH antibody (1:200 dilution, Santa Cruz Boitechnology).

### Mammalian two-hybrid assay

CheckMate Mammalian Two-Hybrid System (Promega) was used following the manufacturer’s instructions. Briefly, Huh-7 hepatoma cells (10^6^ cells per 6 cm dish) were co-transfected with pACT-EPHX1_353–455_ and pBIND-HBSP, pBIND-HBSP_1–47_ or pBIND-HBSP_48–111_ vector with the pG5luc as a reporter plasmid, using Lipofectamine 2000 (Invitrogen) according to the manufacturer’s instructions. Paired empty plasmids pBIND and pACT were used as negative controls. At 48 h post-transfection, the cells were harvested and renilla-normalized firefly luciferase activities were measured using the Dual-Luciferase Reporter Assay System (Promega) following the manufacturer’s instructions. The relative luciferase activities were obtained by comparison to the negative controls (pBIND and pACT), which were set to 1 in each experiment. Each transfection was performed three times, and each time in duplicate. The relative luciferase activities are presented as mean ± SD.

### Recombinant adenoviruses preparation

Recombinant adenoviruses were generated by using AdEasy XL System (Stratagene, La Jolla, CA, USA) following the manufacturer’s instruction. Briefly, plasmid pShuttle-IRES-hrGFP-1-HBSP was generated by inserting HBSP gene into the vector pShuttle-IRES-hrGFP-1. HBSP-expressing (pAdHBSP) or control (pAdControl) recombinant adenoviral vectors were then generated by homologous recombination of pAdEasy-1 and pShuttle-IRES-hrGFP-1-HBSP or pShuttle-IRES-hrGFP-1 in E. coli BJ5183-AD-1. The colonies obtained were screened for appropriate recombination events by Pac I restriction endonuclease analyses. The pAdHBSP and pAdControl were then digested with Pme I and used to transfect the 293A packaging cell line (Invitrogen) to produce HBSP-expressing (HBSP-Ad) and control (GFP-Ad) recombinant adenoviruses. Exponentially growing Huh-7 hepatoma cells were infected with these recombinant adenoviruses at a multiplicity of infection (MOI) of 100. This dose of virus was sufficient to give 100% infectivity as determined by GFP expression under fluorescence microscopy.

### Analysis of effects of HBSP on styrene oxide (STO) hydrolysis

4 μg of mouse liver microsomal protein (Sigma, St. Louis, MO, USA) was incubated with 40 or 100 μg of bacterially expressed NUS-StrepII-tagged HBSP, HBSP_1–47_ (N terminal 47 amino acids of HBSP), HBSP_48–111_ (C terminal 64 amino acids of HBSP) or Nus-StrepII (negative control) [21] for 20 min at 37°C. After incubation, the reaction was initiated by adding 400 μL of 0.1 M potassium phosphate buffer (pH7.4) and 1 mM of STO (Sigma). Then the mixture was incubated for 20 min at 37°C. The reaction was terminated by adding 1 mL of cold ethyl acetate. The mixtures were measured by high performance liquid chromatography (HPLC) as described before [22]. The STO was identified by comparison of their retention times with co-injected authentic standards of STO, and quantified by integrating the areas under the peaks. The assay was performed three times, and the results were expressed as mean ± SD.

### Analysis of effects of HBSP on B[alpha]P metabolism

4 μg of mouse liver microsomal protein was incubated with 40 or 100 μg of StrepII-tagged HBSP, HBSP_1–47_, HBSP_48-111_or NUS-StrepII (negative control) for 20 min at 37°C. After incubation, the reaction was initiated by adding 200 μL of 0.1 M potassium phosphate buffer (pH7.4), 10 μM NADPH (Sigma) and 0.5 μM of B[alpha]P (Sigma). Then the mixtures were incubated for 20 min at 37°C. The reaction was terminated by adding 1 mL of cold ethyl acetate. The mixtures were measured by HPLC as previously described [23]. The B[alpha]P metabolites were identified by comparing their retention times with co-injected authentic standards of B[alpha]P and B[alpha]P-7,8-diol-9,10-epoxide (BPDE) and were quantified by integrating the areas under the peaks. B[alpha]P overall metablic turnover was expressed as percentage of initial substrate concentration. The analyses were performed three times, and the results were expressed as mean ± SD.

### Cell culture and B[alpha]P exposure

Huh-7 hepatoma cells were cultured in Dulbecco’s modified Eagle’s medium (DMEM, Invitrogen) supplemented with 10% fetal bovine serum (FBS, Invitrogen). 5 × 10^5^ Huh-7 hepatoma cells were infected with 100 MOI of recombinant adenoviruses GFP-Ad and HBSP-Ad, respectively. 72 hours after the infection, the cells were treated with 1 μM of B[alpha]P for 24 h, and used for detection of cellular BPDE-DNA by immunocytochemistry. Cells used for assay of proliferation, cell cycle, transformation and tumorigenicity were prepared by viral infection and continuously treated with 1 μM of B[alpha]P for 4 weeks (10 passages), then the cells were harvested and kept at -80°C until use. These cells were designated as Huh-7/HBSP/B[alpha]P (Huh-7 hepatoma cells infected with HBSP-Ad and treated with B[alpha]P), Huh-7/GFP/B[alpha]P (Huh-7 hepatoma cells infected with GFP-Ad and treated with B[alpha]P), Huh-7/HBSP (Huh-7 hepatoma cells infected with HBSP-Ad and without treating by B[alpha]P) or Huh-7/GFP (Huh-7 hepatoma cells infected with GFP-Ad and without treating by B[alpha]P).

### RNA interference assay

100 pmol of either small interfering RNA targeting mEH (mEH siRNA, Santa Cruz Biotechnology) or negative control was used for transfection by lipofectamine RNAiMAX reagent (Invitrogen) in accordance with the manufacturer’s instructions. The cells were collected using RIPA lysis buffer (Pierce) 48 h after transfection. The protein concentration was measured with a BCA Protein Quant Kit (Bio-Rad, Hercules, CA, USA).

### Western blotting analysis

A total of 30 μg protein was subjected to 12% SDS-PAGE, and then electrophoretically transferred to a polyvinylidene fluoride (PVDF) membrane (Millipore, Billerica, MA, USA). Protein blots were incubated separately with a panel of specific antibodies such as anti-mEH (1:500 dilution, Santa Cruz Biotechnology) and anti-β-actin (1:4000 dilution, Sigma). An alkaline phosphatase (AP)-conjugated goat anti-mouse IgG was used as a secondary antibody. CDP-Star reagent (Roche Diagnostics) was used for color development.

### Immunocytochemistry assay of BPDE-DNA

The procedure was performed as previously described [24]. Briefly, when the cells on sterilized glass coverslips reached 70-80% confluence, they were fixed with 4% paraformaldehyde in PBS, and permeabilized with 0.2% Triton X-100 in PBS. After incubation with anti-BPDE-DNA (1:50 dilution, Santa Cruz), the cells were incubated with a biotinylated secondary antibody followed by streptavidin conjugated with horseradish peroxidase (HRP) for 10 min. The immunoreaction was visualized using 3-3′ diaminobenzidine tetrachloride (DAB, Santa Cruz). The slides were mounted with Eukitt (Sigma) and observed with an Olympus BX60 microscope. Images were captured with Image-Pro Express 6.0 (IPE6.0) software.

### Bromodeoxyuridine cell proliferation assay

Huh-7/HBSP/B[alpha]P, Huh-7/GFP/B[alpha]P, Huh-7/HBSP or Huh-7/GFP were seeded triplicate in 96-well plates at 2 × 10^3^/well. Cell proliferation was determined daily for 9 days by bromodeoxyuridine (BrDU) assay according to the manufacturer’s instructions. Briefly, after the cells were plated and serum-starved for 24 hours, BrDU was added to each well at a dilution of 1:2000 and the cells incubated for an additional 24 h. The BrDU incorporation (a measure of DNA synthesis and growth) was measured by using the BrDU Cell Proliferation assay kit (CalBiochem, San Diego, CA, USA) at 450 nm using a microplate reader. The assay was performed three times, and the results were expressed as mean ± SD.

### Cell cycle analysis

Huh-7/HBSP/B[alpha]P or Huh-7/GFP/B[alpha]P were seeded in 6-well plates at 1 × 10^4^/well and serum-starved with 0.5% FBS DMEM for 24 h. Cells were harvested after serum starvation at day 2, 4, and 6 days. Cells were fixed with ice-cold 70% ethanol (pre-chilled at -20°C), washed with PBS (pH 7.2), incubated with 0.05 mg/mL propidium iodide (PI, Sigma) and 1 μg/mL RNase A at 37°C for 30 min in dark. The DNA content of 10,000 cells was analyzed by flow cytometry (Beckman Coulter, Miami, Florida, USA) and CXP 2.2. software. The percentage of each phase of the cell cycle was determined.

### Anchorage-independent growth in soft agar

The assay was performed as previously described [25]. Briefly, 2 × 10^3^ Huh-7/HBSP/B[alpha]P or Huh-7/GFP/B[alpha]P were suspended in 0.3% agarose in DMEM supplemented with 10% FBS, and plated in 60 mm dishes over a basal layer of 0.6% agarose in the same medium. All dishes were incubated at 37°C in a 5% CO_2_ humidified atmosphere, and were examined microscopically for colony formation after a 2-week incubation.

### Tumor xerograft

All the procedures involving animals were approved by Experimental Animal Ethics Committee, Fujian Medical University. The method was used as previously described [26] with minor changes. Briefly, the right flanks of BALB/c nude mice (nu/nu) (male, 4 weeks old) were inoculated subcutaneously with pooled 2 × 10^6^ of Huh-7/HBSP/B[alpha]P or Huh-7/GFP/B[alpha]P in 0.2 mL PBS per animal. Tumor size was measured every 3 days, and the tumor volume was calculated by using the following formula: (length × width^2^)/2. After 21 days of inoculation, all mice were sacrificed, tumors were dissected out, weighted, and processed for immunohistochemistry.

### Immunohistochemistry

Tissues were fixed in 4% neutral buffered formalin, processed, then embedded in paraffin and cut into 5 μm sections. Tissues sections were deparaffinized and rehydrated. Endogenous peroxidase were blocked with 10 min incubation in 3% H_2_O_2_ in PBS. After blocking of non-specific sites with 1.5% blocking serum in PBS for 1 h at room temperature (RT), tissue sections were incubated 1 h at RT with the anti-BPDE-DNA. After a 30 min reaction with a biotinylated secondary antibody, slides were washed with PBS and incubated with streptavidin conjugated with HRP for 10 min. The reaction was then revealed with DAB. Then the slides were mounted with Eukitt and observed with an Olympus BX60 microscope. Images were captured with IPE6.0 software.

### Availability of supporting data

The data sets supporting the results of this article are available in the Addgene plasmid repository, IDs 53113 and 53114, http://www.addgene.org/depositing/70984/

## Results

### In vitro and In vivo interaction between HBSP and mEH

**Figure 1:**
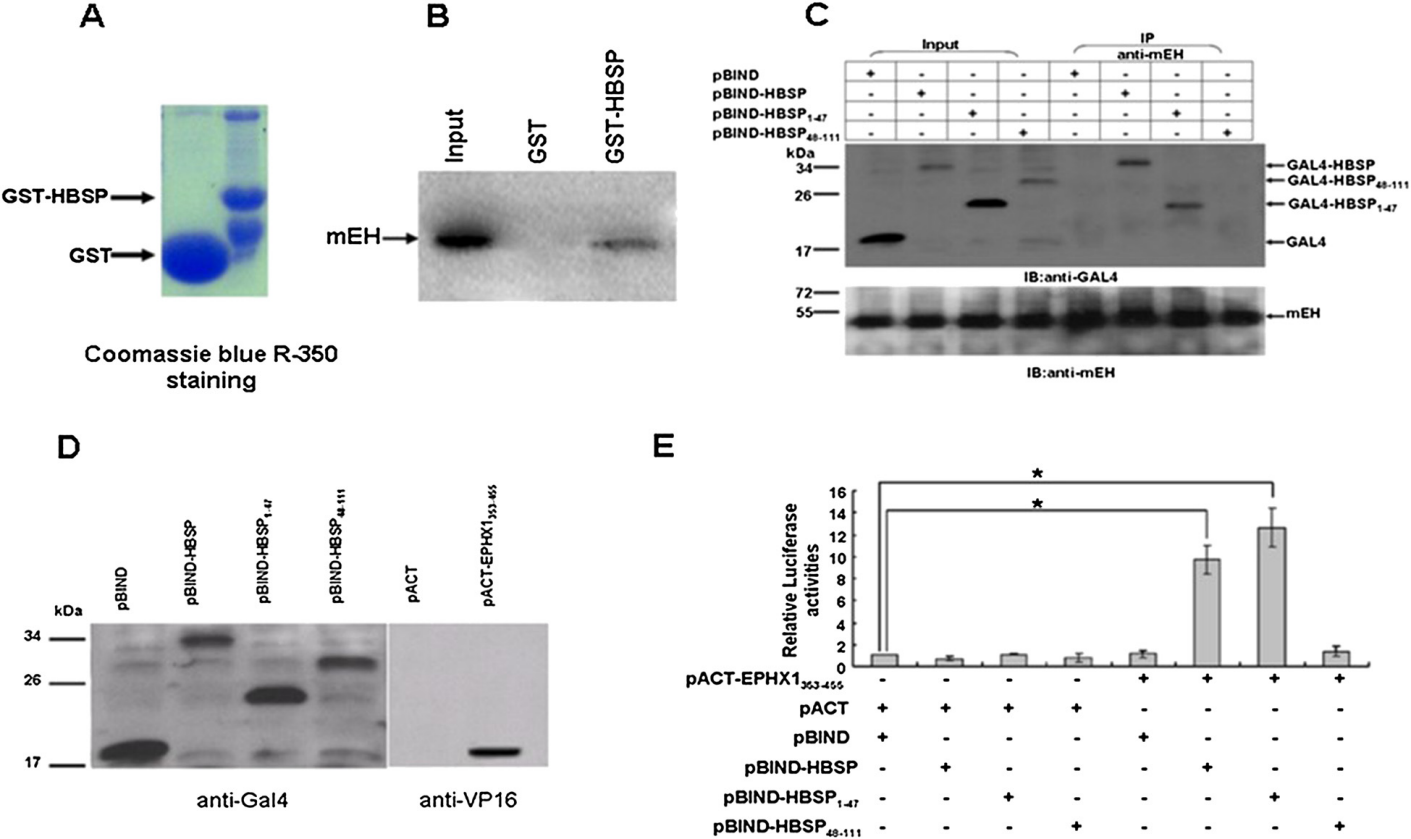
***In vitro *and *In vivo *interaction between HBSP and mEH. (A)** Bacterially expressed GST and GST-HBSP recombinant proteins bound to glutathione-sepharose beads. **(B)** GST pull-down assay. GST recombinant proteins were incubated with ^35^S-labeled mEH, immobilized proteins and subjected to SDS-PAGE and autoradiography. **(C)** Co-IP assay showing the interaction between HBSP and endogenous mEH in Huh-7 hepatoma cells. Cells were separately transfected with pBIND-HBSP, pBIND-HBSP_1–47,_ or pBIND-HBSP_48–111_. Cell lysates from transfected cells were immunoprecipitated with anti-mEH, and the immunoprecipitation complexes were subjected to immunoblotting with anti-GAL4BD (upper) or anti-mEH (lower). **(D)** Western blot analysis of full-length and truncated HBSP, expressed as a fusion protein, with GAL4 DNA binding domain (left). mEH353-455 expressed as a fusion protein, with VP16 activation domain in Huh-7 hepatoma cells (right). **(E)** Mammalian two-hybrid assay. HuH-7 cells were lysed 48 h after transfection and renilla-normalized firefly luciferase activity was determined using the dual luciferase assay system. Data are presented as means ± SD for three independent experiments. The firefly luciferase expression is given as folds over the background (set arbitrarily at 1). (* *P* < 0.01).

A GST pull-down assay was performed to confirm the direct interaction between recombinant HBSP and recombinant mEH in vitro. The result showed that the recombinant GST fusion proteins were well expressed and precipitated by glutathione-Sepharose 4B beads (Figure 1A). GST-HBSP fusion protein complexed with ^35^S-labeled mEH (Figure 1B).

Co-immunoprecipitation (Co-IP) assays were performed to examine the intracellular complex formation between recombinant HBSP and endogenous mEH. As shown in Figure 1C, endogenous mEH in Huh-7 hepatoma cells efficiently co-immunoprecipitated with full-length HBSP and HBSP_1–47_, but not HBSP_48–111_ or GAL4BD, indicating that the mEH-binding site of HBSP is located within the N-terminal half of the molecule (residues 1–47).

A mammalian two-hybrid system was further employed to enable an intracellular evaluation of the binding between HBSP and mEH in Huh-7 hepatoma cells. The results demonstrated that GAL4BD-tagged HBSP, HBSP_1–47_ and HBSP_48–111_ as well as VP16-tagged mEH_353–455_ expressed well in transfected Huh-7 hepatoma cells (Figure 1D). The luciferase activities (Figure 1E) of Huh-7 hepatoma cells co-transfected with pACT-mEH_353–455_ and pBIND-HBSP or pBIND-HBSP_1–47_ were significantly higher (~9 fold for pBIND-HBSP and ~12 fold for pBIND-HBSP_1–47_) as compared to the negative control (P < 0.01), while the luciferase activity for the pBIND-HBSP_48–111_ group was similar to negative control. The results indicate that HBSP-mEH binding interaction can be detected within mammalian cells and that the mEH-binding site of HBSP molecule is again localized to the N-terminal half.

### HBSP enhances the hydrolysis activity of mEH

**Figure 2:**
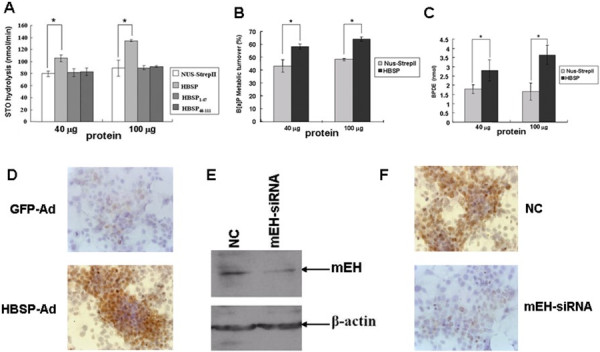
**Effects of HBSP on the hydrolysis activity of mEH. (A)** Effects of HBSP on the hydrolysis of STO. STO was analyzed by HPLC and quantified. The experiments were performed for three times (* *P* < 0.05). **(B-C)** Effects of HBSP on the metabolism of B[alpha]P. B[alpha]P and BPDE were analyzed by HPLC and quantified. The B[alpha]P overall metabolic turnover was expressed as% initial substrate concentration. The experiments were performed for three times. (* *P* < 0.05). **(D-F)** Effects of HBSP on the mEH-dependent BPDE-DNA adduct formation in Huh-7 hepatoma cells. After GFP-Ad or HBSP-Ad infected Huh-7 hepatoma cells were treated with B[alpha]P, cells were submitted for immunocytochemistry assay using anti-BPDE-DNA. Images were taken at × 400 magnification **(D)** After treating with mEH-siRNA and exposed to B[alpha]P, expression level of mEH in Huh-7 hepatoma cells were evaluated by western blot **(E)** and intracellualr BPDE-DNA level was detected by Immunocytochemistry assay. Images were taken at × 400 magnification **(F)**.

Hydrolysis of the styrene oxide (STO) to styrene glycol was widely used to evaluate specifically the hydrolysis activity of mEH in cell free mouse liver microsomes [16]. The results (Figure 2A) showed that hydrolysis of the STO by mouse liver microsomes in the presence of 40 μg and 100 μg HBSP was faster than that in NUS-StrepII (negative control) (105.13 ± 5.62 vs 79.56 ± 4.72 and 134.52 ± 1.66 vs 89.33 ± 13.62, respectively, P < 0.05). In contrast, as for HBSP_1–47_ and HBSP_48–111_, no significant differences were observed (P > 0.05). These observations suggest that HBSP can enhance the hydrolysis activity of mEH, and this effect is dependent on HBSP integrity since neither HBSP_1–47_ nor HBSP_48–111_ was functional.

The influence of HBSP on the metabolism of B[alpha]P in cell-free mouse liver microsomes was also measured. The results demonstrated that the overall turnover of B[alpha]P (expressed as% initial substrate concentration) (Figure 2B) in the presence of 40 μg or 100 μg of HBSP was higher than that of NUS-StrepII (58.65 ± 1.92 vs 43.49 ± 4.78 and 64.48 ± 1.78 vs 48.56 ± 1.0, respectively, P < 0.05). In addition, substantially more BPDE (Figure 2C), was formed in the presence of 40 μg or 100 μg of HBSP as compared to the negative control (2.79 ± 0.58 vs 1.79 ± 0.24 and 3.65 ± 0.52 vs 1.65 ± 0.47, respectively, P < 0.05). These results indicated that HBSP could enhance the metabolism of B[alpha]P in cell-free mouse liver microsomes.

The influence of HBSP on B[alpha]P metabolism in living cells was further investigated. As shown in Figure 2D, the Huh-7 hepatoma cells infected with HBSP-Ad exhibited more intense immunostains than control cells, indicative of higher amount of BPDE-DNA adducts within the nuclei. In addition, when mEH in HBSP-Ad infected cells was knocked down by specific siRNA (Figure 2E), the staining intensity was reverted back to the level similar to the negative control (Figure 2F). These results indicated that HBSP accelerated the metabolism of Benzo[alpha]pyrene through interaction with mEH in Huh-7 hepatoma cells.

### HBSP enhances the proliferation of B[alpha]P-treated Huh-7 hepatoma cells

**Figure 3:**
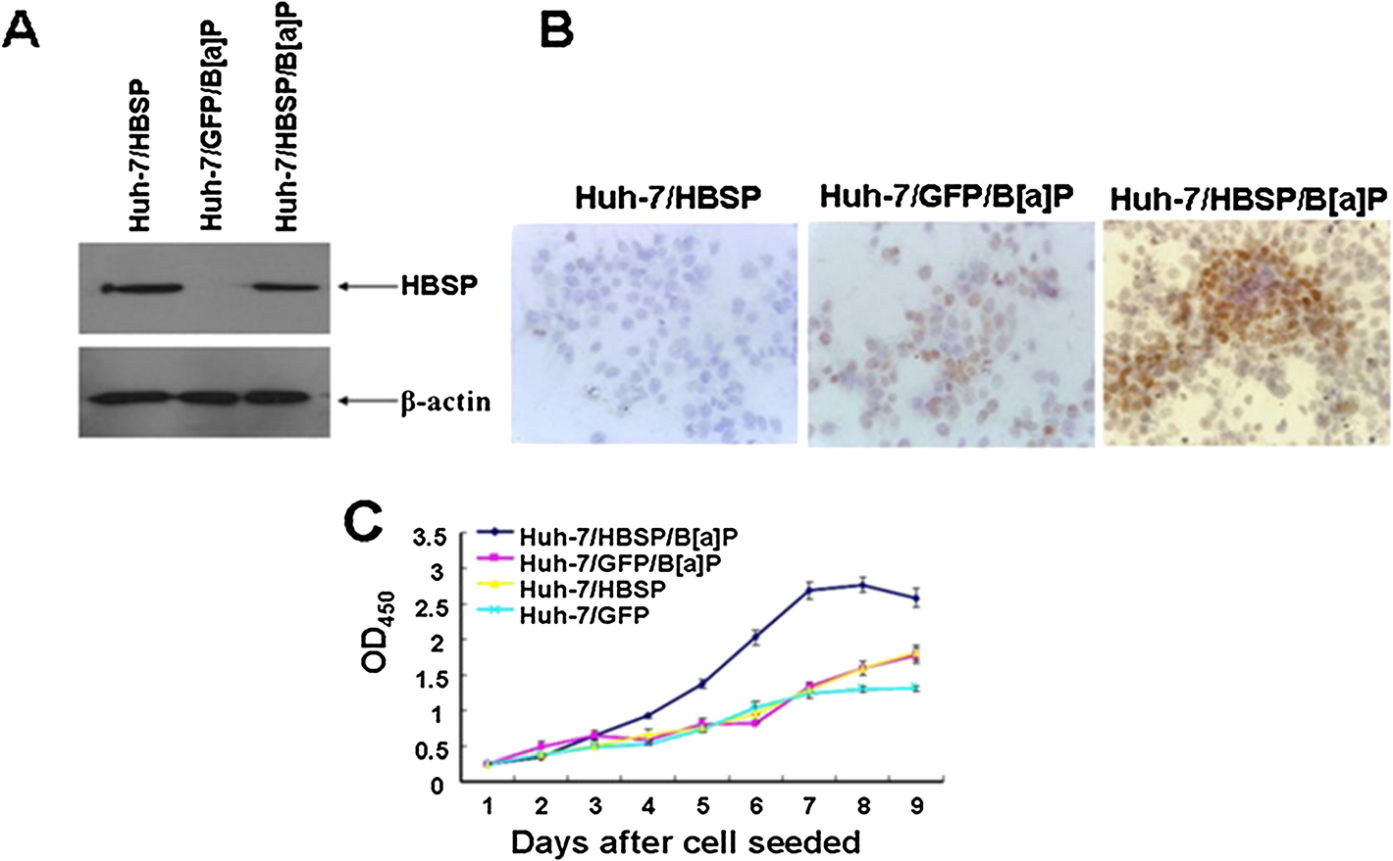
**Effects of HBSP on the proliferation of B[alpha]P-treated Huh-7 hepatoma cells. (A)** HBSP expression in Huh-7/HBSP/B[alpha]P, Huh-7/GFP/B[alpha]P or Huh-7/HBSP. 30 μg of cellular proteins were subjected to 12% SDS-PAGE, transfered to a PVDF membrane, and probed with anti-mEH. β-actin served as a loading control. **(B)** Detection of BPDE-DNA in Huh-7/HBSP/B[alpha]P, Huh-7/GFP/B[alpha]P or Huh-7/HBSP cells. Cells on sterilized glass coverslips were detected for BPDE-DNA by immunocytochemistry assay. Images were taken at × 400 magnification. **(C)** Cell proliferation of Huh-7/HBSP/B[alpha]P, Huh-7/GFP/B[alpha]P, Huh-7/HBSP or Huh-7/GFP cells. Cells were seeded in 96-well plates at 2 × 10^3^/well, cell proliferation was determined daily in triplicate for 9 days by BrdU assay. The optical density (OD) was measured at 450 nm using a microplate reader. The analyses were repeated three times, and the results were expressed as mean ± SD.

As shown in Figure 3A and B, Huh-7/HBSP/B[alpha]P and Huh-7/HBSP could express HBSP and Huh-7/HBSP/B[alpha]P showed a more intense immunoreactivity for anti-BPDE-DNA antibody than Huh-7/GFP/B[alpha]P. In order to investigate the effects of HBSP on the proliferation of B[alpha]P-treated cells, Huh-7/HBSP/B[alpha]P, Huh-7/GFP/B[alpha]P or Huh-7/HBSP cells were seeded and cultured, BrdU assay was performed. The results demonstrated that the proliferation of Huh-7/HBSP/B[alpha]P increased from day 4 to day 7, and significantly faster than that of Huh-7/GFP/B[alpha]P, Huh-7/HBSP or Huh-7/GFP (Figure 3C). These results indicated that HBSP could enhance the proliferation of B[alpha]P-treated Huh-7 hepatoma cells and that this enhancement is not the result of HBSP alone directly acting upon Huh-7 but requires the presence of B[alpha]P.

### HBSP induces cell cycle alterations of B[alpha]P-treated Huh-7 hepatoma cells

**Figure 4:**
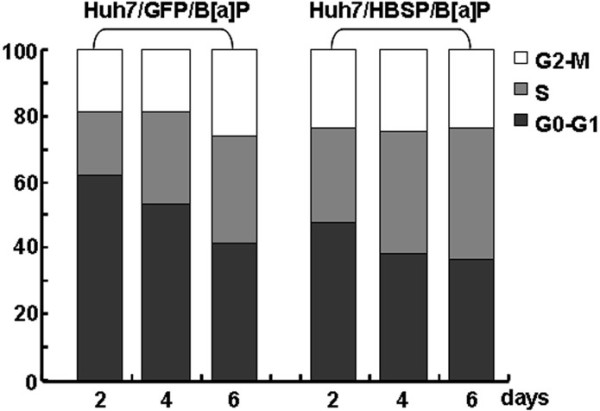
**Effects of HBSP on cell cycle alterations of B[alpha]P-treated Huh-7 hepatoma cells.** 1 × 10^4^ of Huh-7/HBSP/B[alpha]P or Huh-7/GFP/B[alpha]P cells in 6 well plates were serum-starved for 24 h, the cells were harvested at the time of 2, 4, and 6 days after serum starvation. Cells were fixed with ice-cold 70% ethanol (prechilled at -20°C), washed with PBS, incubated with 0.05 mg/ml PI and 1 μg/ml RNase A at 37°C for 30 min in the dark. The DNA content of 10,000 cells was detected by flow cytometry and analyzed by CXP 2.2. software. The percentage of each phase of the cell cycle was determined. The results were one representative data from three independent experiments.

It has been reported that the observed increase in the proliferation of cells treated with B[alpha]P was accompanied by an increased G1/S transition [27,28]. To investigate the effects of HBSP on the cell cycle in B[alpha]P-treated cells, cell cycle phase distributions of Huh-7/HBSP/B[alpha]P and Huh-7/GFP/B[alpha]P were compared at day 2, 4, and 6 after serum starvation. The results showed that Huh-7/HBSP/B[alpha]P displayed a dramatic alteration in cell cycle phase distribution with a decrease in the fraction of the cells in G1 phase and a corresponding increase in the fraction of the cells in S phase (Figure 4). The percentage of S phase in Huh-7/HBSP/B[alpha]P was 45.99%, 57.95% and 58.14% for 2, 4 and 6 day, respectively, significantly higher than those of Huh-7/GFP/B[alpha]P (37.74%, 42.94% and 48.95%, respectively). These data showed that HBSP increased G1/S transition of B[alpha]P-treated Huh-7 hepatoma cells.

### HBSP enhances anchorage-independent growth of B[alpha]P-treated Huh-7 hepatoma cells in soft agar

**Figure 5:**
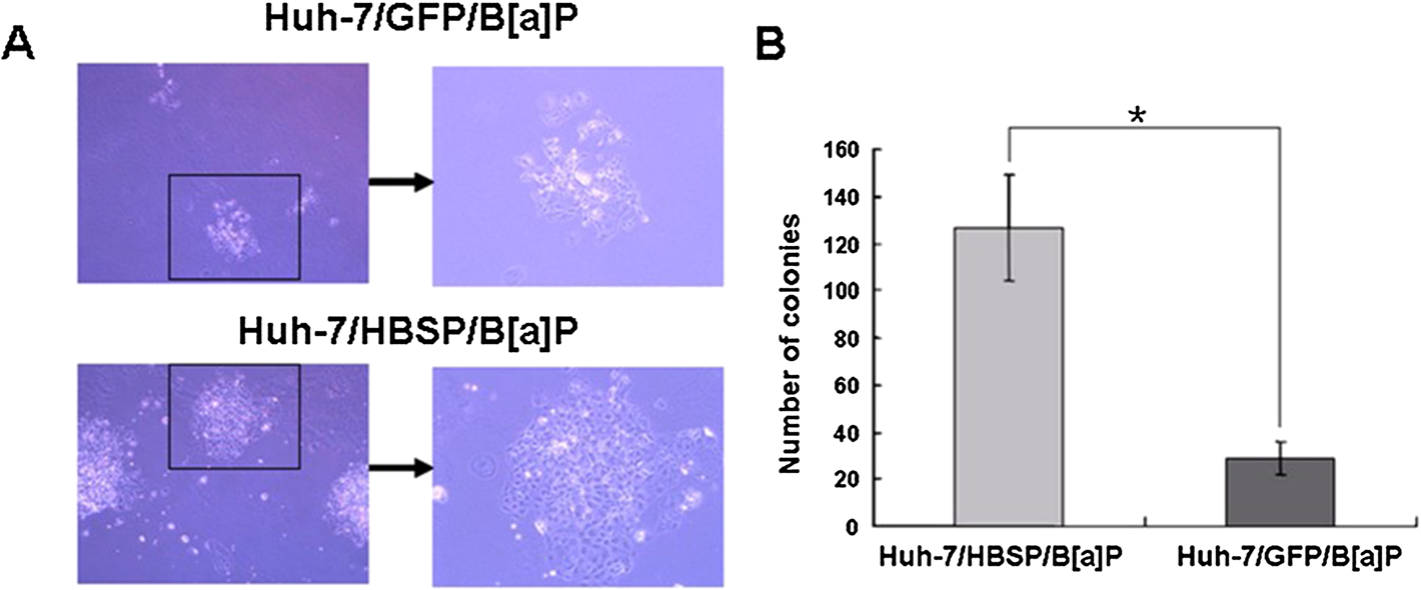
**Effects of HBSP on anchorage-independent growth of B[alpha]P-treated Huh-7 hepatoma cells in soft agar. (A)** Huh-7/HBSP/ B[alpha]P or Huh-7/GFP/ B[alpha]P cells grown in soft agar 14 days after plating. Images on left were taken at × 40 magnification. Images on the right were × 100 magnified and images of the boxed sections depicted on the left. **(B)** The number of colonies grown in the soft agar. (* *P* < 0.05).

To investigate whether HBSP affects the transformation of B[alpha]P-treated Huh-7 cells, the anchorage-independent growth in soft agar was performed. The results revealed that Huh-7/HBSP/B[alpha]P grew significantly better than the control of Huh-7/GFP/B[alpha]P in soft agar (Figure 5A). Moreover, Huh-7/HBSP/B[alpha]P cells showed a dramatic increase in the number of the colonies in soft agar than Huh-7/GFP/ B[alpha]P (Figure 5B). These data indicate that HBSP enhances malignant transformation of B[alpha]P-treated Huh-7 hepatoma cells.

### HBSP enhances tumor development of B[alpha]P-treated Huh-7 hepatoma cells in nude mice

**Figure 6:**
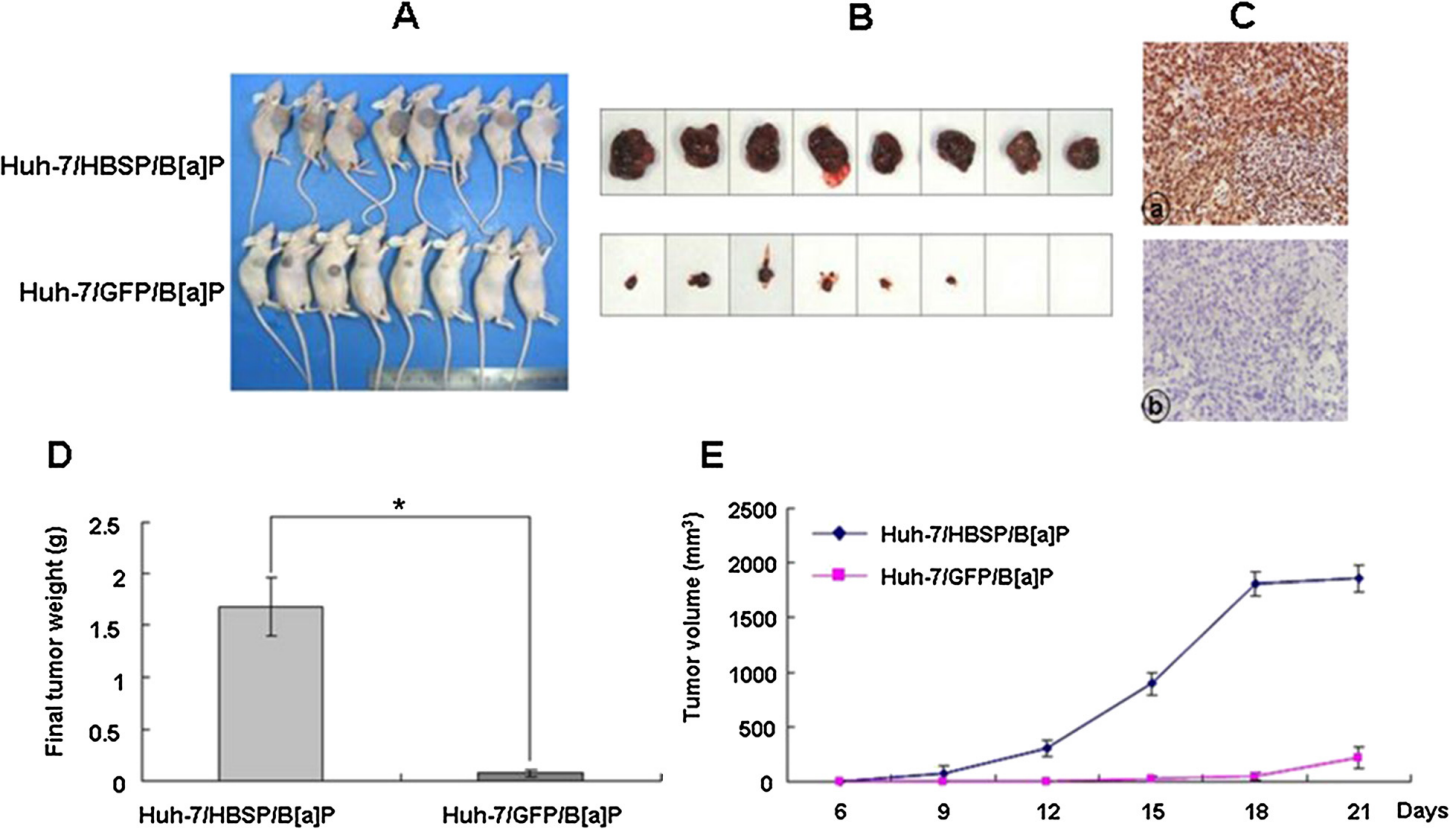
**Effects of HBSP on tumorigenicity of B[alpha]P-treated Huh-7 hepatoma cells in nude mice.** BALB/c nude mice were inoculated with either the Huh-7/HBSP/B[alpha]P or Huh-7/GFP/B[alpha]P. Tumor size was measured every 3 days. All mice were sacrificed after 21 days of inoculation. **(A)** The resulting tumors photographed on the 21st day after inoculation. **(B)** The tumors dissected out from the sacrificed mice. **(C)** BPED-DNA in tumor tissues detected by immunohistochemistry assay. Images were taken at × 400 magnification. **(D)** Weights of tumors dissected out from the sacrificed mice (* *P* < 0.01). **(E)** Tumor volume during growth, results were expressed as mean ± SD.

To test the effect of HBSP on tumorigenicity of B[alpha]P-treated Huh-7 cells, nude mice were inoculated with Huh-7/HBSP/B[alpha]P or Huh-7/GFP/B[alpha]P. All of the mice inoculated with the Huh-7/HBSP/B[alpha]P developed visible tumors within 9 days after the injection, while only six of eight (75%) mice inoculated with Huh-7/GFP/B[alpha]P developed visible tumors on day 15 post-injection. After the mice were sacrificed on day 21 post inoculation (Figure 6A), the xenografts were dissected out (Figure 6B). The volume and weight of the xenografts were measured, and the presence of BPDE-DNA in the xenografts was detected. The results demonstrated that there was more intense BPDE-DNA staining in the xenografts derived from the Huh-7/HBSP/B[alpha]P as compared to that in the xenografts from Huh-7/GFP/B[alpha]P (Figure 6C). In addition, xenografts derived from the Huh-7/HBSP/B[alpha]P cells were significantly larger and heavier compared to the those from the Huh-7/GFP/B[alpha]P cells (xenografts volume: 1861 ± 120 mm^3^ vs 222 ± 98 mm^3^; xenografts weight: 1.69 ± 0.28 g vs 0.08 ± 0.04 g, P < 0.01) (Figure 6D and E). These data suggest that HBSP enhances tumor development of B[alpha]P-treated Huh-7 hepatoma cells, and this enhancement of HBSP is accompanied by the formation of more BPDE-DNA adducts in the tumor cells.

## Discussion

Virus-chemical interactions as mechanisms of carcinogenesis have been proposed based on epidemiological investigations. For example, tar-based carcinogens generated from lysol virgin dunching, cigarette smoking, or cooking over burning wood were co-carcinogens for human papillomavirus (HPV) types 16 and 18 in cervical cancer [29-33]. Nitrite inhalants and aluminosilicates were considered as co-carcinogens for human herpes virus type 8 (HHV-8) in oncogenesis of human immunodeficiency virus (HIV)-associated Kaposi’s sarcoma (KS) [34,35]. Hepatocarcinogenesis involves a complex interplay of viral, environmental, and host factors. Long incubation period between the time of initial HBV infection and the onset of HCC suggests that HBV plays a role in liver oncogenesis as a cofactor or tumor promoter rather than a direct cause of cancer [36-38]. In fact, the crucial role of hepatocarcinogen diethylnitrosamine in the tumor development of HBx transgenic mice [39], and the synergistic interactions between HBV and aflatoxins or alcohol in HCC have been well documented [40,41]. In the present study, HBV-B[alpha]P interaction in hepatocarcinogenesis was further confirmed and the mechanisms explored.

B[alpha]P is a major toxicant in diesel exhaust, charcoal-broiled food, industrial waste byproducts, and cigarette smoke [42,43]. Administration of B[alpha]P by different routes has been shown to result in the production of tumors in several species of animals [44,45]. The metabolic activation pathway of B[alpha]P involves three sequential enzymatically catalyzed reactions including monooxygenation by cytochrome P450 (CYP) enzymes, hydration by mEH and a second CYP-catalyzed oxidation to generate a carcinotoxic ultimate product, BPDE [46], which is capable of binding covalently to DNA in the living cells to form adducts [16]. In the present study, it was demonstrated that HBSP generated by a 2.2 kb singly-spliced HBV pre-genomic RNA could specifically bind to the C-terminal region (amino acid residues 353–455) of mEH, and resulted in an increase in the hydrolysis activity of mEH, as demonstrated by hydrolysis assay using STO, an artificial substrate. In addition, it was also demonstrated that HBSP could accelerate B[alpha]P metabolism in mouse liver microsomes leading to an increased amount of BPDE-DNA adducts. In Huh-7 hepatoma cells, expression of HBSP resulted in an increased formation of BPDE-DNA adducts, and this enhancement effect could be blocked when mEH was knocked down by siRNA, implicating an indispensable mechanistic role that mEH plays in the enhancement. HBSP begins at the HBV polymerase start codon and contains the first 47 amino acids residues of polymerase (Met1-Asn47, HBSP_1–47_), followed by the 64 amino acids residues (Glu48-Tyr111, HBSP_48–111_) resulting from a frameshift event [10,21]. More importantly, nucleotide sequence alignment comparison among the eight (A-H) known genotypes of HBV showed that the sequence following the 5′-splice site at nucleotide 2447 and that following the 3′-splice site at nucleotide 489 are highly conserved (data not shown), suggesting that generation and function of HBSP are HBV genotype-independent. In this study, it was shown that while HBSP-mEH complex formation was mediated by N terminal 47 amino acid residues of HBSP, the enhancement of mEH activities requires the intact HBSP molecule.

B[alpha]P accelerates cell cycle progression from G1 phase to S phase and induces cell proliferation in human embryo lung fibroblasts (HELF). This effect was mediated by c-Jun activation by PI-3 K/Atk/ERK signaling pathway with the up-regulation of expression of cyclin D1, E2F1 and pRb [47]. Moreover, BPDE has been documented to promote cell transformation and tumorigenesis through the induction of cyclin D1 via the PI-3 K /Akt/MAPK and p70s6k-dependent pathway [48]. Recently, interaction of HBSP with CTSB was found to promote hepatoma cell motility and invasion, the mechanisms involve the secretion and activation of proteolytic enzymes, increased tumor-induced angiogenesis, and activation of the MAPK/Akt signaling [49]. In the present study, it was demonstrated that HBSP could enhance the cell proliferation, accelerate the G1/S transition, and promote cell transformation and tumorigenesis of B[alpha]P-treated Huh-7 hepatoma cells. These results suggest the co-carcinogenesis of HBSP and B[alpha]P in HCC, and shed a new light for potential therapeutic intervention to prevent hepatocarcinogenesis in the hepatitis B patients with high B[alpha]P exposure by mEH and/or HBSP suppression treatment.

## Conclusions

In summary, the results of this study demonstrate that HBSP promotes carcinogenic effects of B[alpha]P through enhancing the hydrolysis activities of mEH, thereby accelerating the conversion of B[alpha]P to BPDE and increasing the formation of BPDE-DNA adducts while the physiological significance of HBSP remains to be verified by comparing full-length HBV (capable of replication and expression of all the viral proteins at appropriate ratios) with a mutant incapable of splicing or expression of the intact HBSP. These findings not only broaden the knowledge of virus-chemical interactions in co-carcinogenesis of HCC, may also lead to the further study of chemoprevention of HBV-associated HCC.

## Competing interests

All authors declare that they have no competing interests.

## Authors’ contributions

JYC, WNC, BYJ and WSL performed the experiments, interpreted the results. YLW and LLL contributed to the scientific discussion. JYC drafted the manuscript. XL was the project leader. All authors read and approved the final manuscript.

## Pre-publication history

The pre-publication history for this paper can be accessed here:

http://www.biomedcentral.com/1471-2407/14/282/prepub
